# Can research integrity prevail in the market? Lessons from commissioned research organizations

**DOI:** 10.1080/08989621.2021.1937603

**Published:** 2021-06-09

**Authors:** Knut Jørgen Vie

**Affiliations:** Work Research Institute, Oslo Metropolitan University, Oslo, Norway

**Keywords:** Research integrity, research ethics, commissioned research, competition, research bias

## Abstract

Competition and exposure to market forces can make it difficult for researchers to conduct their work with integrity. Some research organizations must acquire most of their funding through commissioned research, providing research services for paying clients. Studying such organizations can give insight into how researchers try, and sometimes fail, to balance academic norms with the need to secure funding. Based on interviews with social scientists in commissioned research organizations, this study shows how clients can exert an undue influence on the research process and how competition for funding can make it difficult to live up to academic quality standards. However, it also shows how commissioned research can be a source of identity and motivation. It involves a high degree of impact and access to good data, as clients commission research projects because they want knowledge to solve specific problems. Moreover, the participants discussed how they and the organizations where they worked learned from their experiences how to counteract the negative aspects of competition.

## Introduction

Misconduct, bias, and conflicts of interest pose a significant threat to the trustworthiness of research. The literature on these issues often points to competition for goods such as funding and permanent positions as reasons for unwanted research practices (Bouter 2020). Understanding such mechanisms is therefore crucial when it comes to promoting research integrity. A common assumption in studies of research integrity is that research takes place at universities. While most studies recognize that research organizations face increasing competition, they still tend to treat researchers as engaged in self-initiated basic research, in addition to teaching and administrative tasks. However, universities are not the only institutions with research as their primary purpose. Other forms of research organizations exist. Private companies can have in-house research teams, and some of these companies have research as their main product. Other examples of non-university research organizations are *public institutes, government laboratories, research and technology organizations, contract research organizations*, and *research institutes* (Fisher and Kalbaugh 2013; Gulbrandsen 2011). These organizations must typically secure most of their funding from external sources, particularly under competition, in the form of *commissioned research*.

While there is no universally accepted definition of commissioned research, the following proposal provide a good starting point:

Research conducted for pay, where the financer want research conducted on a specific topic or problem, and where the research is not undertaken in the direct employment of the financer^1^ (Holden and Fløtten 2018, 7)

Under these conditions researchers must compete with other researchers for funding in a market, and research projects are initiated by external actors rather than the researchers themselves. For their organizations to stay economically viable, somebody must be willing to pay for their work. As the funders of research, clients have a position of power which they can abuse in order to exert an undue influence on the research process or publication of the findings (Ham 1999). This worry is covered by the *European Code of Conduct for Research Integrity* which states that the following is an unwanted process: “Allowing funders/sponsors to jeopardise independence in the research process or reporting of results so as to introduce or promulgate bias” (ALLEA 2017). Studying *commissioned research organizations* where external clients provide most of the funding is a good starting point for generating insight into how competition for funding can introduce bias and conflict of interest, or even lead to misconduct and other questionable practices.

The present study reports on interviews with social scientists in research organizations that historically have secured most of their funding through selling research services. It draws on the following research question: how are the conditions for conducting research with integrity at commissioned research organizations? This question is approached through the lens of neo-institutional theory (Greenwood et al. 2008), treating these organizations as hybrid, as they must balance academic norms with the logic of the market. This approach allows for identifying challenges that researchers under a high degree of market pressure can face in their day-to-day work and the practices they develop to deal with such pressure. The study is an extension of a focus group study conducted by the Horizon2020-project PRINTEGER^2^ which looked at how researchers in general conceive of research integrity and the mechanisms they believe that can threaten it (Kennedy et al. 2018).

### Commissioned research

Funding for research comes in several different forms. For the most part, universities receive their funding directly from the government or in the form of students paying tuition. Other forms of funding include commissioned research and sponsored research. These terms are sometimes used interchangeably and sometimes to denote two different types of funding. In Norway, where the present study was conducted, *oppdragsforskning*, which is typically translated as commissioned research, is discussed separately from *bidragsforskning* which is typically translated as sponsored research (Holden and Tone 2018).

Under this classification, most sponsored research takes the form of philanthropy or grants from external funding agencies. While sponsored research is acquired through competition with other researchers, who must write funding applications or appeal to philanthropists, it differs from commissioned research in important ways. Firstly, while funding agencies create calls for research on specific topics, they do not provide research funding because they need knowledge for solving a specific problem that they themselves have. Consequently, they do not have the same interests and stakes in the results. Secondly, researchers who apply for funding from sponsors are typically the originators of the ideas for the specific project, within the topic determined by the call. In commissioned research on the other hand, clients have more control over the project as they are the originators of the idea behind it. Sponsored research involves some of the same risks as commissioned research, such as bias and disagreements over the ownership of research results. However, the power relationship is even more unbalanced in favor of the funder in commissioned research on account of their interests in the results and the fact that the client is the source of the project idea.

While differentiating between commissioned and sponsored research in this way is an established approach in Norway, it does not translate well to an international context, where the two terms at times are used in overlapping ways. For example, *industry sponsored research* is used as a label in clinical research, where it refers to industry actors who pay research organizations to conduct research on a particular topic or to conduct clinical trials. Here, the funders have a high stake in the results, which results in bias (Lundh et al. 2017). In Norway, this would be labeled commissioned research, but the biomedical research community uses the label sponsored research. Therefore, it could be prudent to use terms that are more descriptive of the phenomenon to which they refer. As the risk of bias in external funding mainly stems from the client’s interests in the results and the fact that the client is the originator of the project idea, differentiating between *researcher initiated* and *funder initiated* externally funded research would capture the relevant differences between the two forms of funding. However, the present paper will use the term commissioned research as defined above since the research context is Norwegian and this was the label the participants used on their own work.

While commissioned research have certain similarities with consultancy, it is important to underscore how the two differ. Consultants are hired for solving specific problems or providing knowledge on a specific topic, but they rarely do so with the same commitment to research norms as commissioned researchers have. Commissioned research is still research, which demands training in research methodologies and adherence to the ethos of research. On the one hand, the legitimacy provided by an adherence to the disinterested modes of knowledge production research aspire toward can give a competitive advantage over consultants. On the other hand, commitment to research norms can be a competitive disadvantage as rigorous research can be expensive.

Internationally, countries differ in how they organize their research sectors. Therefore, it is difficult to obtain a comprehensive picture of how many researchers are engaged in commissioned research and how many commissioned research organizations exist. OECD (2020) statistics show that about 250000 researchers in their member states work at nonprofit organizations and governmental organizations outside of higher education. This number indicates that a significant amount of research takes place outside universities, but it does not give insight into the nature of this research. There exist a handful of studies on commissioned research, but these are limited to the role of independent research institutes in knowledge production and innovation in technical and scientific fields, existing alongside the higher education sector (Gulbrandsen 2008, 2011; Hallonsten 2017; Late 2019).

The present study focuses on commissioned research organizations in Norway. According to NIFU, independent research institutes conduct research for 14.8 billion NOK in 2018 and produced 9385 full-time worker equivalents that year, divided on 138 different organizations.^3^ This research constitutes roughly 20% of R&D spending in Norway, including the private sector. This statistic has recently become somewhat less comprehensive. Several independent research institutes have recently merged with universities, where they keep much of their independence and still must secure their own funding themselves but fall outside the statistics.

The commissioned research organizations based in the social sciences in Norway primarily work on three different topics, (1) international development and peace, (2) the welfare state, and (3) regional development (The Research Council of Norway 2017). The practice of commissioned research at these organizations is characterized by a need to acquire funding from external sources, primarily through channels for public procurement or from private businesses. Research typically takes the form of projects with a limited timeframe where one or more researchers work together. The final product is typically a report to the client.

Researchers in commissioned research are expected to compete in a knowledge market against consultants and other analysts, but also against university researchers engaged in more basic forms of research. They must provide relevant applied research while also facing the challenges of providing rigorous methods and analyses. The Research Council of Norway recognizes this challenge in a report on independent research institutes oriented toward the social sciences.

Many of the institutes experience a squeeze between on the one hand the growing expectations of government that the universities should undertake commissioned research in areas that the institutes historically have viewed as their own and on the other hand commercial consultants, whose capacity to address commissioned research has been increasing. The result is a pressure for the institutes to demonstrate their superiority in terms both of ability to address user needs and to do so on the basis of research that is scientifically strong (2017, 2)

This quote shows that these organizations are put under increased competition. University researchers are increasingly expected to engage in commissioned research, while consultants are increasingly adopting research as a mode of knowledge production. This increased competition comes on top of their already highly competitive situation, where they must compete with other commissioned research organizations for projects. This makes studying commissioned research organizations a fruitful approach for producing insight into the effects of competition on the ability of researchers to do their work with integrity and free from bias and conflicts of interest. The fact that universities are increasingly seeking external funding in the form of commissioned research makes this work even more important, as they can benefit from learning of the experiences of organizations who have operated in this field for decades.

### Research integrity in commissioned research

The study of research integrity is an ongoing endeavor, and what it means to have integrity in research is discussed continuously (Kuroki 2018; Shaw 2018). According to one definition, research integrity is “the performance of research to the highest standards of professionalism and rigor, in an ethically robust manner” (Hiney 2015, 3). The literature tends to label serious deviations from research integrity as misconduct, which it typically divides between two categories (Horbach and Halffman 2017). The first includes fabrication and falsification of data, along with plagiarism, while the includes *questionable research practices* and consists of other practices that have the potential to distort the scientific record without necessarily constituting outright fraud. There exist many studies of academic research, comparatively fewer of commissioned research, and almost none of research integrity in commissioned research. Regarding the latter, one of the few examples is a mixed-methods study in Norway, which found that commissioned research is, for the most part, conducted according to acceptable standards (Kaiser et al. 2003). This result is surprising since the research integrity literature concludes that competition leads to increased risk of misconduct. In addition to this report, two studies have outlined the authors’ personal experiences with commissioned research while working at universities. According to Ham (1999), the client’s control over the research findings can come into conflict with academic freedom and the researcher’s need to publish on the findings. Clients can also misrepresent the findings, especially if they are not published. Richter and Hostettler (2015) point to neoliberalism and temporary assignments as driving forces behind the increased demand for securing external funding. They have personally experienced clients who unduly tried to make them change their findings to put the clients in a better light.

The literature on research integrity points to many different factors that can promote the likelihood of research misconduct or bias. Some studies look at individual factors like personality (Antes et al. 2007; Tijdink et al. 2016) or career stage (Fanelli et al. 2019). Other studies point to systemic factors, such as competition and a situation where one must *publish or perish* (Davis, Riske-Morris, and Diaz 2007; Martin 2013; Oravec 2017; Tijdink et al. 2016). Such pressures can lead to corner-cutting behavior or unethical practices (Macfarlane 2009) or even an erosion of research norms as such (Edwards and Roy 2017; Macfarlane 2019). At the organizational level, competition can also harm the work environment by distorting workplace relations (Anderson et al. 2007), leading to sabotage, secrecy, and less efficient research. Competition to secure funding, an essential component in commissioned research, is also described as a risk in the literature (Drenth 2006; Resnik 2014; Yang 2013). The potential negative influence of external funding has been particularly salient in biomedical fields, where industry funded clinical trials have been found to have a higher chance of positive results (Als-Nielsen et al. 2003). This indicates that external funding can lead to bias.

### Commissioned research as a hybrid practice

Universities have undergone a vast transformation in recent decades. Marginson (2000) talks about four transformational processes: (a) globalization, (b) decline of government funding, (c) emergence of professionalized management and slippage of collegial ideals, and (d) tendencies to the deconstruction of the academic profession. Research integrity needs to be understood and studied in the context of such transformations, especially the decline in funding and hence exposure to market forces, as the research integrity literature finds that perverse incentives increase the risk of misconduct (Bouter 2020).

In line with Gulbrandsen (2011), this study treats commissioned research institutes as *hybrid organizations*. They operate in multiple organizational fields, which impose different sets of expectations, norms, and “rules of the game” (Thornton, Ocasio, and Lounsbury 2012). Using the vocabulary from neo-institutional theory, this approach conceives hybridity as consisting of competing *institutional logics*, i.e. “institutionalized templates for organizing” and “a field’s shared understanding of the goals to be pursued and how they are to be pursued” (Battilana, Leca, and Boxenbaum 2009, 68). According to Friedland and Alford (1991), ideal-type institutional logics are the market, the corporation, the professions, the state, the family, and religion.

Two of these logics, the market, and the professions, are central for commissioned research organizations. On the one hand, an ideal market logic involves a template wherein researchers must produce research that is relevant enough for somebody to be willing to pay for it to keep their institutes economically sustainable. The logic of the market ideally means that high-quality research is produced in the dynamic relation between supply and demand, where the “best” researchers will win the commissions and hence produce adequate knowledge to the clients. The ideal-type market logic thereby has many connotations with consultancy work.

On the other hand, academic logics connects with the logic of the professions. This template treats academics as a classic profession (Fox and Braxton 1994), i.e. with high status and a high degree of professional autonomy (academic freedom), even though one may also claim that different academic disciplines constitute a heterogeneous (Lamont 2009) – and perhaps even incommensurable (Kuhn 2012) – field. Therefore, it is prudent to speak of academic logics and professions in the plural, emphasizing various types of academic standards. Professional norms and quality assessments are based primarily on social control, such as peer review and other forms of organized skepticism (Merton 1973).

Table 1 summarizes key elements of the two ideal logics. Commissioned research must incorporate elements of both these oppositional logics and is thus a hybrid practice. These two logics are ideal types, and the real picture is blurrier, as the different types of organizations are converging. For example, universities face increasing market pressure, and several studies have therefore conceptualized them as hybrid organizations with competing institutional logics (Frølich et al. 2013; Grossi, Dobija, and Strzelczyk 2019; Guarini, Magli, and Francesconi 2020). Lepori (2016) goes so far as to state that universities are prime examples of this type of organization.

**Table 1.** Characteristics of the two logics.

	Market logic	Professional/academic logics
Key actors	Consultants, analysts	Academic researchers
Type of research	Applied research	Basic and applied research
Key template for knowledge production	Relevance	Rigor
Knowledge dissemination	Limited, trade-secrets	Publication, teaching
Quality assessment	Purchasing procedures	Peer-review
Funding	Competition in the market	Traditionally dominantly in-house

Despite this increasing convergence, commissioned research organizations have a unique form of hybridity in the need for securing funding through selling research. What is particularly important in the analysis is that the notion of research integrity may look different within each logic. The study assumed that the two types of logics may conflict with each other. Of particular interest was whether the market logic in this form of research could disturb academic logics to such an extent that it becomes a threat to research integrity. This framework allows the study of commissioned research as something structurally different from traditional research, where traditional research to a greater degree is placed in academic logics. In comparison, commissioned research involves a greater degree of contradiction and hybridity.

## Methodology and data

### Project background

The present study is a continuation of a focus group study on research integrity conducted as part of the Horizon2020-project PRINTEGER, which had as its goal to study research integrity as it is practiced by researchers in their day-to-day work. The members of the PRINTEGER-project developed a research protocol, under coordination of the PRINTEGER-team at the University of Bristol. The Bristol team also piloted the interview questions The project took the form of a small-scale, exploratory, qualitative research project (Ritchie et al. 2013). Focus group interviews were conducted in the UK, Italy, Estonia and Norway. The interviews resulted in a deliverable in the project (Kennedy et al. 2018).

The author of the present paper conducted the four Norwegian focus group interviews together with a colleague, where three groups included researchers at three different levels of seniority, and the fourth group included participants in administrative positions. For the Norwegian focus groups, we reached out by e-mail to all the researchers at five different departments and institutes with different research profiles. However, with the exception of a PhD student in the group consisting of junior researchers, only potential participants from an institute oriented toward commissioned research in the social sciences volunteered for the three groups containing researchers. As the researchers were from different disciplines within the social sciences, we decided that the groups were diverse enough to proceed with the interviews.

### The follow-up interviews

In the focus groups, the participants pointed to challenges resulting from the competitive nature of commissioned research that made it difficult to do their jobs with integrity. As the literature on research integrity has neglected commissioned research organizations, the author of the present paper initiated a follow-up study to explore this topic further through one-on-one interviews, which the present paper reports on. The three Norwegian focus group interviews with researchers formed a basis for making minor changes to the interview guide from the PRINTEGER-project to focus more directly on the nature of commissioned research. These three interviews are also included as the data used in the present paper. The revised interview guide is attached to this paper as an appendix. The interview guide begins with a warm-up question regarding what constitutes good research, and subsequently transitions into the most important questions for this paper which relate to the researchers’ understanding of research integrity and barriers and challenges to integrity as they themselves understand that term.^4^

### Recruitment

The members of the Norwegian PRINTEGER-team worked at an institute oriented toward commissioned research themselves^5^ and therefore knew of researchers who had faced challenges of the kind found in the focus group. While threats toward research integrity are unfortunately not rare, it is rare enough to justify a purposive approach to sampling for the sake of efficiency. Therefore, the recruitment started with some of these researchers. The data gathering expanded outside of this department by including commissioned researchers and leaders who had discussed integrity challenges in the media and researchers whom the other interviewees believed had experience integrity challenges. The participants were asked to participate via e-mail. The e-mail included a letter with information on the topic of the interview and the aims of the study, along with information on anonymization, data protection, and the rights of the participants. Only one of the researchers asked opted not to participate. The data gathering proceeded in this fashion until few new perspectives emerged in the interviews, having reached saturation.

In sum, 28 researchers and directors from commissioned research organizations participated in the present study. These participants came from five different commissioned research organizations oriented toward the social sciences, with the majority recruited from two of them. 17 participated in one-on-one interviews, while the remaining 11 participated in the focus groups. Additionally, the focus group consisting of junior researchers included a PhD student which did not come from a commissioned research organization. We conducted the focus groups in the first half of 2017, and the author of the present paper conducted the one-on-one interviews in the first half of 2019. The participants were only interviewed once. A list of the experience level, gender, and distribution among the participating organizations can be found as an appendix in Table 2. Due to the sensitive nature of the topic they are not numbered and linked to the quotes presented in the findings to reduce the risk of identification.

**Table 2.** Overview of interview participants.

Position	Gender	Organization	Interview type
PhD student	Female	A	Focus group A
PhD student	Female	F (noncommissioned research organizations)	Focus group A
Senior researcher	Female	A	Focus group A
Senior researcher	Male	A	Focus group B
Senior researcher	Female	A	Focus group B
Senior researcher	Female	A	Focus group B
Senior researcher	Female	A	Focus group B
Research professor	Male	A	Focus group C
Research professor	Female	A	Focus group C
Research professor	Female	A	Focus group C
Research professor	Female	A	Focus group C
Research professor	Female	A	Focus group C
Institute director	Male	A	One-on-one
Research professor	Male	A	One-on-one
Senior researcher	Male	A	One-on-one
Institute director	Female	B	One-on-one
Institute director	Male	C	One-on-one
Senior researcher	Female	D	One-on-one
Research professor	Female	E	One-on-one
Research professor	Male	E	One-on-one
Research professor	Male	E	One-on-one
Research professor	Male	E	One-on-one
Research professor	Male	E	One-on-one
Senior researcher	Female	E	One-on-one
Senior researcher	Female	E	One-on-one
Senior researcher	Male	E	One-on-one
Senior researcher	Male	E	One-on-one
Researcher	Male	E	One-on-one
Researcher	Male	E	One-on-one

### Interviews, transcription, and analysis

The interviews were recorded and transcribed verbatim, except for two that were transcribed only in part by request of the participants due to the topic’s sensitive nature. Most of the interviews were conducted face-to-face, while two were conducted over Skype. In order to safeguard anonymity, care was taken to ensure that the interviews were not observed by colleagues of the participants, for the most part by conducting them at a meeting room on another floor than where the participants worked. The focus groups all lasted about 1 hour and 45 minutes. The one-on-one interviews typically lasted 45 minutes, while a few lasted 1 hour and 30 minutes.

The interviews were analyzed thematically (Creswell 2014) using NVivo. The author coded the data alone. The coding was inductive in that the goal was to identify challenges and practices in an understudied field. It was also inductive in that the researchers were allowed to define integrity and associated terms. The analysis initially coded the interviews in accordance with the questions they answered in the interview guide, where each question constituted its own node. In this way, the participants’ understanding of topics such as the nature of commissioned research and barriers to conducting research with integrity under such conditions were identified. During this process, various themes emerged, such as the various practices developed by the researchers and the organizations where they worked. The coding was especially sensitive to issues that the participants described as particular to commissioned research compared with research found at universities or the work conducted in consultancy.

### Ethics approval and reflexivity

The focus group study received research ethics approval from the Faculty of Health Sciences Research Ethics Committee at the University of Bristol. The follow-up interviews were reported to the Norwegian Center for Research Data. All the participants signed consent forms. During the data gathering the author of the present paper was employed as a PhD-student, a temporary position, at the department where most of the one-on-one interviews were conducted. This constitutes a risk of bias, as these participants were his seniors in that organizations and the organization constituted a potential further employer. Research integrity can be a sensitive topic, and a reputational risk for both those interviewed and the organizations where they work. The author’s lack of seniority risks reluctance to discuss difficulties in conducting research with integrity. This risk was mitigated by conducting interviews with participants outside that organizations, and the findings from all the organizations revolved around the same themes indicating that the participants from the different organizations had the same level of willingness to be open.

According to Jackall (1988), getting access to reputationally risky stories through interviews takes significant experience and familiarity with the field. However, in the present case the participants were more eager to talk about difficulties regarding producing high-quality research with integrity in research than the author expected. Possibly, they saw the interview as an opportunity to contribute to improving the conditions they work under by making problems known. While the author did not work in commissioned research projects himself, working at an organization where this has historically been the most important source of funding made it easier to identify and pursue interesting topics during the interviews.

## Findings

Overall, the study finds that researchers in commissioned research can struggle with explicit and implicit undue influences from their clients. Furthermore, their need to continually chase funding can lead to sloppiness and quality issues, which the participants experienced as matters of integrity and ethics. In this way, the responses show how competition can be a threat to integrity in research. However, the participants also discussed how researchers working under competition can engage actively with the challenges they encounter and create practices to help them deal with and prevent difficult situations. Moreover, commissioned research can have positive aspects. It offers high-impact projects and good access to data. Some participants said that they would rather work in commissioned research than work in a university setting with stable government funding. For these researchers, the market aspect of their work was a source of motivation and identity. This section will elaborate on each of these findings.

### Handling undue influence from difficult clients

A central theme in the interviews involved being in a subordinate power relation with clients due to the need to secure further funding. Alienating clients could make them reluctant to commission further projects. As one participant stated, “You can get burned you know. You can write in a way that makes a client uninterested in financing you” (researcher). “Getting burned” here refers to the risk that the researcher will not get access to further projects on account of having presented negative or unflattering findings regarding the client. Another participant said that he had heard from a leader, “Several times, that you have to remember who pays us, pays our salary” (researcher). The leader in this case was worried that the researcher was about to present findings which would make him unpopular with the client.

As commissioned research depends on clients for staying economically viable, undue influence can put researchers in difficult situations where following research norms such as honesty can get them into trouble. Clients can exert undue influence in different ways. One way this power was manifest was in clients seeking to change or suppress research results or designs directly.

Another challenge is when the client does not like what he gets. And we have experienced that. Either wanting us not to publish or asking us to reconsider. Things like that. We have experienced that. And a final variant is the one where there are results where … Where we get a phone call from a lawyer […] And then you have to take a stand and be sure that you have systems you can point to (institute director)

The systems referenced here are quality assurance routines and codes of conduct. Referring to such systems can put weight and legitimacy behind a researcher’s attempt to stand up to a difficult client. Another example mentioned by a researcher was that clients can “blacklist” researchers that they do not like, and the participant used a client essential to the institute’s economic viability as an example.

You cannot, in a way, say that they are not doing their job […] And they are not the only ones you cannot get on the bad side of […] I know, for example, that there are researchers at our institute who are on a kind of informal blacklist (senior researcher)

In this case, the influence is indirect. The client is not trying to intervene in the research process directly but is rather relying on an implicit threat of blacklisting researchers who should not receive further funding, in order to keep researchers from publishing results that are embarrassing for the client.

Clients can also potentially influence researchers and their results in a more subtle way. A research director reflected on being careful not to “sit on the client’s lap” and adopt the client’s worldview. This risk was seen as especially high when doing research on a new topic. In commissioned research, it can be necessary to work on several different topics throughout one’s career, as funding for one topic can periodically dry up.

One should be very careful not to get on the lap of the client and lose sight of the fact that they often can want to pull the project in a particular direction […] and from time to time you get too close […] We have seen that sometimes when people have approached new topics they can rely too much on the client when they describe the problem […] And then we have to ask ourselves whether we are entirely sure that this is the right approach to describing it (institute director)

This quote highlight factors that can limit the critical distance between the client and researcher required by academic norms. Interestingly, both getting too close and too distant was seen by this director as a failure to achieve the necessary critical distance. Researcher who are too far removed from the client and topic in question struggle to see the influence exerted on them on account of insufficient knowledge and experience. Distance is thus not necessarily the same as critical distance. Some researchers reported that the room for conducting critical research was significantly narrower in institutes oriented toward commissioned research.

Well, you cannot, in a way, be an institute researcher^6^ with a very critical fundamental agenda. You know, you can have critical conclusions in projects about how things are done and the like. But, you know, when I started at [the institute] I became an inclusion researcher. Before I started at [the institute], I was an oppression researcher. And that is the price you pay (senior researcher)

Before joining a commissioned research organization this researcher described the system in question as oppressive of a protected group. After becoming a commissioned researcher, the organizations he had previously described in critical terms became his clients, and he therefore felt that he had to reframe his work in positive terms, as a matter of including the marginalized group, in order not to alienate his clients.

### Misuse of research results by clients and potential for negative impact

Typically, clients commission research because they want to use it for a specific purpose. One challenge mentioned by the researchers was the risk of being misused by clients in a way that threatens research integrity. Allowing oneself to be exploited by clients who want specific results can lead to an unethical impact in the real world if the results in question are biased. It can also damage one’s credibility as a researcher if somebody gets the impression that one is a “lackey” of one’s clients, as one research professor phrased it.

The following excerpt is an example of where the participant realized that he was probably being exploited:

Sometimes you can feel like you were deceived, you know. By the client or something like that. I have one such case. Where you are supposed to do a project, and you participate in a planning group, and I think that they knew […] that this can be used in a kind of power struggle (researcher)

While the cases above describe a situation where the participant experienced that the client had dubious intentions before and during the research project, other participants described the risk of misunderstandings or misuse after finishing the project. There is a risk that their work may be presented, interpreted, or used in a skewed or even disingenuous way.

I got hold of the summary from a journalist who had received it. And there was a presentation of our report which did not fit what we believed was in the report, so [the ministry] got a completely different picture of our research than we had presented in our summary (senior researcher)

While misuse and misrepresentation of research occur in most fields, the participant felt a special responsibility for ensuring that commissioned research is represented truthfully due to its applied nature and the direct relationship with the users of the research. If the research is used to inform policy, as in the quote above, the policy risks wasting resources or even having a negative impact on the problem in question if the research is not interpreted properly. The participants saw this as a matter of integrity as their names were used to support the position of the client.

### Research quality under pressure

Time pressure and worries about research quality are ubiquitous in all research sectors. However, according to the participants, commissioned research involves more time pressure than most other types of research due to contractual obligations and competition in acquiring projects. Projects must be finished within a specific timeframe, and researchers must seek further funding while working on the projects they have already acquired. The following quote shows how researchers can experience this pressure as very demanding:

At the most, I have had twelve projects going on simultaneously because I have had many small projects, and then it is self-evident that you cannot give it your best effort anywhere really. And that gives you an enormous discomfort, you know, it materializes physically almost in the form of stomach pains (senior researcher)

Small projects allow very little time for doing research of sufficient quality. However, the quoted researcher felt that taking such projects was necessary in order to reach her expected earnings, putting her in a difficult situation where she had to balance multiple projects at the same time. She felt that she was unable to perform at an adequate level in all of them at the same time. This resulted in a feeling of discomfort and guilty conscience which was strong enough to manifest in physical pains.

When research is conducted for pay, delivering low-quality results can be experienced as unethical or disingenuous, especially if one delivers less than promised or if the client plans on applying the research. As one researcher put it, “Well, sloppiness can glide over into the unethical […] it is sold as research, as science. And bad science can be fraudulent, but it is not done in bad faith. It is just incompetence, sloppiness, or tight deadlines” (institute director). Low quality research is here described as functionally equivalent with fraud, as both fraud and low-quality research can be wrong and have similar negative consequences if applied in solving problems. Sloppiness can take many forms. The informants mentioned examples typically categorized as questionable research practices in the literature, like analyzing and presenting results based on data they knew were of too low quality, interviewing fewer people than promised, and making too hasty generalizations.

Another challenge related to limited time involved not being able to follow the most current developments in their respective disciplines, which in turn can make it challenging to produce good research, thus making it difficult to live up to academic norms. As one of the researchers stated, “Much of the interesting things you have to … You have to do that in your spare time, you know. As I was told by a leader […] Reading, you know, research literature – that is something we do in our spare time” (research professor). Producing high-quality research is difficult if there is little room for reading, if quality is equated with keeping up with and contributing to the newest developments in the field.

### Becoming a good commissioned researcher through socialization

In several of the interviews, questions about integrity were initially met with responses that it was simply about making good choices. As one participant put it, “What matters is just to, you know, manage to take a stand, and trust that you have done a good enough job” (senior researcher). After a few questions however, and some reflection, the researchers revealed that there are mechanisms in play aimed at preserving integrity, where some are aimed at the issues particular to commissioned research.

Since commissioned research organizations in Norway are subject to the same laws regulating data management and research ethics as other research organizations, systems such as codes of conduct, research ethics approval, and protection of sensitive data were discussed in the interviews. The participants were lukewarm regarding these measures. Some found them overly bureaucratic and misaligned with the practice of research, and a few condemned these systems using strong language. Others found that the introduction of formal systems had increased ethical awareness and rooted out sloppy practices regarding data protection. As the present paper is about commissioned research and not the Norwegian research ethics system in general, this section will focus on the measures and mechanisms that the participants believed to counteract issues such as undue influence from clients and difficulties in producing high quality research.

According to the participants, taking a stand in a difficult situation, such as standing up to a difficult client, ideally comes after having engaged with a set of learning mechanisms. As one participant stated, “I think if you are experienced, you learn how to […] Yeah, you just have to handle the financer’s expectations in some way. And I feel that … With time, I feel like we have a handle on it” (research professor). Handling clients is here seen as a skill that can be improved with experience. Several of the participants said that teaming new researchers up with more experience researchers was an active policy.

Yeah, we are doing teaching. Training. Of the young. We are doing it. You know, in the way that we put them in projects with those with experience […] And that is a kind of learning process about - how should you approach clients? How should you approach the client’s organization – the work place he represents? The others you meet there? And how should you disseminate the results? (research professor)

By putting younger researchers in teams with those with more experience, they can observe the more tacit aspects of handling situations where one’s integrity is potentially in play. While formal and informal teaching of young researchers happens at most research organization, the responses here indicate that this is a conscious effort in commissioned research, and this teaching is oriented toward issues that are prevalent at institutions oriented toward commissioned research, like handling clients.

One participant said that consultancy was used as a pedagogical contrast when he started in commissioned research, “I have learned a lot from my older colleagues”. These older colleagues signaled things like, “We do not adjust ourselves to the needs of the financiers”, unlike consultants “ … who will write anything” (senior researcher). Teaching is therefore not just a matter of ensuring that new hires are able to handle the difficulties of commissioned research. It is also a way of socializing researchers into commissioned research and its norms regarding ethics and quality.

According to the participants, teams are not just useful for teaching new researchers how to preserve their integrity. They can also promote integrity more directly.

I think that it is important to close ranks internally, that we have each other’s back and everything. That is very important, especially in commissioned research […] you can stand in very difficult situations. And then we must, in a way, be loyal towards each other internally, and we must find ways to handle things externally. Because if you do not have that security at home […] then it is simply quite dangerous to go out there (senior researcher)

According to this researcher, in order to stand up to the pressure exerted by financiers it is important have allies that will back you. The quote also paints lack of internal support as a barrier to doing the right thing. Teams are not just described as important for maintaining a minimum of integrity. Working in teams was also seen as a method of quality assurance as researchers vary in their knowledge and experiences. By working together it is possible to paint a more complete picture of the problem the team is studying.

### Using contracts as leverage

An important measure for preserving integrity, was the use of contracts aimed at ensuring academic freedom and the right to publish the results.

We have as a policy not to accept consultancy contracts, just because with those we have no guarantee that the report will be published. So, we always want it to be a part of the contract that we are going to publish based on it (senior researcher)

Consultancy contracts here refer to contracts where researchers commit themselves to doing a specific task, where the client gets control over the results and their dissemination. Ownership of the results of research projects in commissioned research is a topic where several dilemmas can arise. Researchers are committed to academic norms like openness, and this is incompatible with a client’s wish to keep certain findings secret if they for example are uncomfortable or inconvenient. Rather than making this a matter of negotiation between the individual researchers and a client, this has been raised to the level of policy at the organizational level. Good contracts can be used to resolve disputes with the client. As one participant said, “Yeah, for me, the contract is the entire … It is the lifebuoy […] I have read it very closely, and I am, you know, very oriented toward what is says […] I love the contract” (research professor). If a financer for example demands more work than initially agreed, or try to interfere in the research process, pointing to the contract can be an efficient way to settle the matter.

### Reframing success in research

While the researchers reported difficulties in keeping up with academic norms regarding quality, they also argued that exposure to competition allowed them to do high-impact research and get good access to data. As one researcher stated: “And you can say that by replying to calls at [an online public procurement portal] you are very relevant for society because the projects there are about very real issues that they want to be studied” (senior researcher). According to this view, commissioned research has certain merits compared with academic research that can compensate for the difference in academic rigor. As the researchers argued that difficulties in living up to academic norms and standards could be a matter of integrity, aspects of their work that can weigh up for this are, by extension, a matter of integrity as well. Some researchers pointed to relevance as motivational and something that can make working in commissioned research attractive.

The basis is that in commissioned research, you have a client. You have somebody who is interested in the immediate results of what you are doing. And that is something that is very motivating for us who have been engaged in commissioned research in all our years and feel that it is more exciting and meaningful than the classical research conducted at universities (research professor)

While they may not always be up to date on developments in their disciplines, their research has a high impact and is interesting to specific actors, which can be rewarding and motivate them to endure the more demanding aspects of their work.

## Discussion

The researchers gave multiple examples of situations where they had to struggle to balance the demands of professional academic norms with the logic of the market and where the researchers felt that integrity was at stake. The challenges that the researchers believed to be particular to or more prevalent in commissioned research fit into three categories: undue influence from clients, difficulties in producing high-quality research, and the potential negative effects of flawed results if the client puts them to use

The participants presented the various perceived challenges as interacting and having compounding effects. For example, they described how the difficulties in keeping up with academic developments due to underfunded projects and lack of funding for learning were compounded by the need to spread one’s efforts over several different topics and projects, which happens when funding for one topic become constricted. This can lead to an insufficient understanding of a topic, making it difficult to take a critical perspective, as one director noted. A complete account of all such connections is beyond this paper’s scope, but it is important to keep in mind that moral life is complex and that any attempt at reducing itsacrifices much of this complexity (Pincoffs 1986). While the researchers’ perception that operating in a market context can be a threat toward integrity is not a new finding in itself, the interviews give interesting insights into how this can manifest itself in practice, in the researchers’ day-to-day work, with risk factors tightly linked with the market logic found in commissioned research.

The hybrid nature of commissioned research seems to have contributed to a blurry understanding of the relationship between research ethics, integrity, and quality. The researchers described situations where they felt that issues related to two or all three were in play simultaneously. For example, delivering a low-quality product to a client could also be a matter of ethics and integrity. If the product is of lower quality than promised, this can be disingenuous, which is a matter of integrity in the sense that the research is presented as more or better than it is. Dishonesty of this type can also be a form of unethical research. If the client applies low-quality research results, this can have a negative impact, and the literature typically discusses negative consequences of research as a matter of research ethics. While outright fraud saw little discussion in the interviews, low-quality research can be functionally equivalent to fraud, as they could both lead to negative consequences.

While it can be fruitful to discuss the definitions of research integrity, research ethics, and research quality separately, such conceptual work does not always translate easily into the real world, where the concepts are sometimes thoroughly entangled. Others have also observed how these categories can get mixed (Israel 2015), but what is novel about the findings here is that the market logic can make matters of quality into matters of integrity and ethics. Therefore, some concerns regarding ethics, integrity, and quality seem more salient for researchers in commissioned research than for research more firmly planted in the academic logic.

This point is particularly evident regarding quality. An interesting phenomenon in the data was the emphasis on the merits of commissioned research compared with more academic university research, presented above under the heading *Reframing success in research*. While the interviews revealed that researchers in commissioned research sometimes have a hard time living up to the highest academic quality standards and norms, indicating that the hybridity in this form of research has negative aspects, the researchers also pointed out that their exposure to the market gave them high impact and good access to interesting data. In this way, they were able to produce good research on their own terms.

Some of the researchers labeled themselves as “commissioned researchers”^7^ or “institute researchers”,^8^ signaling commitments to both the logics under which they labor, indicating that they have established a sub-identity. They are researchers, but of a specific type, with some idiosyncratic norms, advantages, and disadvantages, where the market logic becomes something positive, a source of identity and motivation. Having multiple sources of identity in an organization can be a source of conflict (Foreman and Whetten 2002). The introduction of a market logic in research is often discussed critically (Macfarlane 2019). However, it seems that the market logic combined with the academic logic can take the form of *positive hybridity* (Fossestøl et al. 2015). Blends and compromises between logics in research can turn into new hybrid practice models, which in turn can mature and become institutionalized (Lepori 2016). In the present case, it seems that researchers at commissioned research organizations, or at least the ones where the interviews took place, have redefined what counts as good research as a way of coping with the difficulties in living up to both the professional logics which provides them legitimacy and the market logic which provides the material conditions they need for maintaining their organizations.

Furthermore, commissioned researchers and the organizations where they work are not passive with regards to the conditions under which they work. The participants discussed several different mechanisms that counteracted the difficulties resulting from competition. Some of these functioned on the personal or team level. Handling difficult clients was described as a skill that can be developed with experience. Knowledge about specific clients and topics was also described as necessary for ensuring the appropriate critical distance. The organizations where the participants work actively tried to institutionalize this experience and knowledge. They did this by teaming experience researchers up with less experienced researchers so that projects would have the necessary capacity to stand up against undue influence, and so that younger researchers could learn from role models how to become a good commissioned researcher. While the participants were lukewarm, and sometimes even hostile, toward external formal systems for research ethics and data protection, they actively used their own quality assurance systems, guidelines, and standardized contracts as leverage against difficult clients. It is likely that their own internal systems were a better fit for the needs of commissioned research, as they were developed in that context.

The findings have some policy relevance. Several of the challenges the researchers pointed out were also identified in the few studies on commissioned research at universities (Ham 1999; Richter and Hostettler 2015). Therefore, the present study’s identification of how researchers experience such challenges can be relevant for organizations that have historically seen less exposure to the market logic. It also shows how integrity issues can interact with each other in a complex way, which is something research leaders and policymakers should be aware of to better reflect on the potential ripple effects of their work. In practice, if policymakers want to mitigate threats toward integrity in commissioned research, a place to begin would be requiring public procurement contracts to include funding for quality assurance and review of the relevant literature. In the cases included in this paper where the researchers felt that they had fallen short of integrity standards, they gave a lack of support from leaders as an important reason, which indicates that cultivating supportive leadership is another measure that could help.

In the interviews, the researchers discussed various practices they had developed locally for ensuring integrity is maintained. However, they described these practices more as a matter of skill than introducing specific measures. Rather than attempting to turn these lessons into policy, it could be prudent for research organizations that have recently started pursuing commissioned research to invite or hire experienced commissioned researchers in order to learn the craft. While the participants described quality assurance systems where the researchers give each other feedback in teams, these systems are only as good as the researchers who participate in them.

The paper has some limitations that can serve as pointers toward further study. It does not aim for representativity, as it is exploratory and reports the experiences of a limited number of researchers. Further research could use the findings as a basis for studying the prevalence of the challenges described by the participants. While the participants discussed difficult situations, a potential limitation is that research on ethical topics risks *social desirability bias*, as participants can engage in reputation management or self-deception when discussing their own ethical conduct (Randall and Fernandes 1991). It is therefore possible that relevant experiences or details were left out by the participants. Studying whether the research and reports produced by commissioned research organizations tends toward a higher level of positive findings as have been done in biomedical fields could be a fruitful avenue to studying the influence of competition on bias further. Further research could also look at commissioned research organizations oriented toward science and engineering to study whether such fields face similar challenges. A prevalent theme discussed only briefly is the changing nature of commissioned research. The interviewees reported experiencing increasing pressure to live up to academic norms at the cost of market norms. This trend is the opposite of what more academic organizations experience, making it an interesting potential avenue of study.

## Concluding remarks

The study finds that the hybrid nature of commissioned research can lead to challenges toward research integrity that are linked tightly with the market logic found in this form of research. Clients can try to intervene directly in research results, which researchers naturally experience as challenging and uncomfortable. They can also exert more subtle forms of control that can lead to bias. While the participants reported that the market logic provided challenges, as expected, they also noted that it provided their work with meaning and identity, as they experienced their work to be highly relevant and impactful due to their clients’ willingness to pay money for it. This relevance can function as a sort of compensation for the difficulties in living up to the highest academic quality standards, which the participants felt that the competition made it difficult to achieve. The participants also discussed how experience can make it easier to deal with competition in a good way, and the organizations where they worked had developed practices for disseminating such experience. Researchers and research organizations are this not passive victims to the forces of competition, but active participants in counteracting such forces. Research organizations with less experience with competition should learn from the organizations who have operated in this field for decades.

## Notes

1. Translated from Norwegian by the author

2. The project website: https://printeger.eu/

3. This website gives further information on the statistics with visualizations. It is available in English, but only using automatic translation: https://www.nifu.no/fou-statistiske/fou-statistikk/instituttsektoren/

4. According to the research protocol, researchers were supposed to be sorted by seniority based on how long ago they had done their Ph.D. However, this was a poor fit for the organizations where the interviews took place. Some of them had traditions where the researchers did their Ph.D. later in their careers than what is usual at universities. Therefore, the presentation of the data refers to them by their academic titles. In Norway, the English titles in these institutions are, in descending order: “research professor”, “senior researcher” and “researcher”

5. This institute is not the same as the one where the participants in the focus groups worked

6. Institute researcher here refers to researchers who work at an independent research institute

7. “Oppdragsforsker” in Norwegian

8. “Instituttforsker” in Norwegian, referring to the sector of independent research institutes

## Appendix

**Semi-structured interview guide**

This guide is adapted to the context of commissioned research from the research protocol for conducting the focus group interviews in the PRINTEGER-project.

**Introduction/warm-up**

- What makes good research?

**Transition question**

- What would you consider as bad research? What would you consider misconduct in research?

**Key questions**

- **Defining integrity**
  - How would you define integrity in research?
  - How do you discuss integrity questions at your place of work?
- **Research culture: expectation and leadership**
  - What barriers and challenges do you think researchers in commissioned research face that can affect research integrity or lead researchers to cheat?
  - Can you describe a situation where you, as a commissioned researcher, can face contradicting expectations?
- **Knowledge, training, and guidelines**
  - Research must comply with multiple guidelines aimed at promoting integrity. How does your workplace try to ensure that researchers are familiar with and act according to such guidelines?
  - Are these kinds of guidelines useful?
- **Support, interpretation, and translation of guidelines**
  - Where have you learned about standards for good research and integrity?
  - Where do you believe researchers in general learn about such standards?
  - What routines do you have for following up integrity issues at your place of work?
- **What works and what can be improved**
  - What do you believe commissioned research needs in order to build and preserve integrity in research?
  - What works at your place of work?
- **Final question**
  - Among the things we have discussed so far, what is the most important focus in order to ensure the integrity of commissioned research?
  - Do you feel that we have covered the most important aspects of research integrity, or is there something you would like to add?
